# Patient-specific sequencing panels enable sensitive circulating tumor DNA analysis in rhabdomyosarcoma independent of genetic profile

**DOI:** 10.1038/s41698-025-01147-6

**Published:** 2025-10-21

**Authors:** Ida Rahmqvist, Agnes Dahlstrand Rudin, Elisabeth Mellström, Raghda R. Ibrahim, Daniel Andersson, Fani Pujol Calderón, Anna Ordqvist Redfors, Niki Rostamzadeh, Wilma Franssila, Christin Karlsson, Fanny Zetterlund, Robert Khashan, Hanna Frostdahl, Tobias Österlund, Torben Ek, Henrik Fagman, Anders Ståhlberg, Martin Dalin

**Affiliations:** 1 Sahlgrenska Center for Cancer Research, Department of Pediatrics, Institute of Clinical Sciences, Sahlgrenska Academy at University of Gothenburg, Gothenburg, Sweden; 2 Wallenberg Centre for Molecular and Translational Medicine, University of Gothenburg, Gothenburg, Sweden; 3 Childhood Cancer Centre, Queen Silvia Children’s Hospital, Sahlgrenska University Hospital, Gothenburg, Sweden; 4 Sahlgrenska Center for Cancer Research, Department of Laboratory Medicine, Institute of Biomedicine, Sahlgrenska Academy at University of Gothenburg, Gothenburg, Sweden; 5 Department of Pediatrics, Queen Silvia Children’s Hospital, Sahlgrenska University Hospital, Gothenburg, Sweden; 6 Department of Clinical Genetics and Genomics, Sahlgrenska University Hospital, Region Västra Götaland, Gothenburg, Sweden; 7 Department of Laboratory Medicine, Institute of Biomedicine, Sahlgrenska Academy at University of Gothenburg, Gothenburg, Sweden; 8 Department of Clinical Pathology, Sahlgrenska University Hospital, Gothenburg, Sweden; 9 SciLifeLab, Institute of Biomedicine, University of Gothenburg, Gothenburg, Sweden

**Keywords:** Prognostic markers, Paediatric cancer

## Abstract

No liquid biomarkers are available for monitoring rhabdomyosarcoma, and treatment evaluation is limited to imaging examinations. Circulating tumor DNA (ctDNA) is a promising disease marker in various malignancies, but generalized ctDNA assays targeting recurrent mutations are unsuitable for childhood sarcomas due to genetic heterogeneity. We developed tumor-informed sequencing panels targeting ten single-nucleotide variants per patient and performed ultrasensitive ctDNA analysis of 130 plasma samples in twelve children with rhabdomyosarcoma. Levels of ctDNA correlated with tumor burden, decreased gradually and became undetectable with successful treatment. All four disease relapses and the one case of primary resistant disease were associated with increased ctDNA levels. In one patient, ctDNA was repeatedly positive during five months before clinical relapse. In contrast, all samples collected during follow-up in patients without relapse were ctDNA-negative. Our findings show that ctDNA, analyzed using a tumor-informed approach, is a sensitive and specific biomarker for rhabdomyosarcoma, also for patients lacking recurrent genetic alterations.

## Introduction

Rhabdomyosarcoma (RMS) is the most common pediatric soft tissue sarcoma, with an annual incidence of ~4.5 cases per million children worldwide^1,2^. RMS is characterized by neoplastic cells with some features of skeletal muscle differentiation and occurs in various anatomical locations, most commonly head and neck, genitourinary organs or extremities^3^. Based on their clinicopathological and molecular genetic characteristics, the World Health Organization currently classifies RMS into four distinct subtypes: embryonal, alveolar, spindle cell/sclerosing, and pleomorphic RMS^4^. Stratification of RMS patients based on clinical, pathological, and molecular features has enabled tailoring of therapy to improve the outcome of poor-prognosis patients and reduce treatment toxicity in patients with less aggressive disease^5,6^. However, while radically resected embryonal RMS localized to favorable anatomical sites is associated with high survival rates (90% relapse-free survival), the outcome for patients with metastatic disease is still dismal (three-year survival rates of 25–30%)^2,7^. Moreover, depending on disease risk classification, 30% of all RMS patients experience disease recurrence after primary treatment, with only 20–30% surviving despite undergoing intense multimodal therapy^8,9^.

Monitoring of treatment response and disease relapse in RMS is currently based on imaging techniques, such as magnetic resonance imaging (MRI) and computed tomography (CT). However, frequent radiology examinations are associated with long-term complications due to radiation exposure or repeated general anesthesia, and tumor size alone may not accurately represent the burden of disease. So far, no clinically implemented biochemical biomarkers are available for disease monitoring in children with RMS.

Circulating tumor DNA (ctDNA) consists of genetic material released into circulation through apoptosis, necrosis or active secretion by cancer cells^10^. Monitoring of ctDNA in various body fluids has emerged as a promising method for assessing tumor burden and treatment response in multiple cancer types^11,12^. ctDNA assays are generally designed to detect recurrent mutations, however, this strategy is difficult in RMS, as such mutations are only present in a subset of cases^13^. Alveolar RMS is characterized by *PAX3/PAX7::FOXO1* fusions, and assays targeting these alterations have proved efficient in monitoring of ctDNA levels in fusion-positive patients^14–18^. Targeted gene panels based on copy number variations (CNVs), other recurrent translocations, and single nucleotide variants (SNVs) have been utilized in an attempt to monitor ctDNA also in the fusion-negative patient group, with the limitation of low sensitivity^15,17–19^.

In this study, we developed customized sequencing panels targeting ten SNVs and utilized them to assess ctDNA levels at various timepoints before, during, and after treatment in twelve children diagnosed with RMS. The level of ctDNA at diagnosis correlated with disease burden, and all four relapses were associated with increased ctDNA levels before or at the time of clinical detection. Our findings emphasize the potential of ctDNA, evaluated using a tumor-informed approach, as a valuable tumor marker for clinical applications in RMS.

## Results

### Patient characteristics

The study included twelve children diagnosed with RMS at a median age of eight years and six months. Ten patients had localized tumors, and two had metastatic disease at diagnosis. Tumor histologies included six embryonal, four alveolar (one with neurogenic component/ectomesenchymoma), and two spindle cell cases. All cases were assessed for *FOXO1A* fusions and *MYOD1* mutations during routine diagnostic workup. Clinical tumor genetic analysis revealed three cases with the *PAX3::FOXO1A* fusion gene, one case with *PAX7::FOXO1A*, and two cases with the *MYOD1* p.L122R mutation (Table 1).

**Table 1:** Patient characteristics

Patient	Age (y)^a^	Sex	Subtype^b^	Localization	Metastatic disease at diagnosis	Treatment protocol^c^	Genetic alterations^d^
C001	9	m	Embryonal	Left cheek	No	CWS, SR, subgroup C	-
C002	8	f	Spindle cell	Left temporal region	No	CWS, HR, subgroup E/CWS 2007 HR/CWS ACCTIVE/RIST	MYOD1 p.L122R
C003	1	m	Embryonal (botryoid)	Floor urinary bladder	No	CWS, SR, subgroup D	-
C032	10	f	Alveolar	Nasal cavity/ ethmoidal cells	No	CWS, HR, subgroup G	*PAX3/FOX01A*
C047	15	m	Embryonal	Left testis	No	CWS, SR, subgroup C	
C068	12	m	Spindle cell/Sclerosing	Below the left ear	No	CWS, HR, subgroup E	*MYOD1* p.L122R
C076	15	m	Embryonal	Scrotum	No	CWS, SR, subgroup B	-
C077	4	f	Alveolar	Left side anterior chest wall	No	CWS, HR, subgroup G	*PAX3/FOX01A*
C090	4	f	Embryonal	Right cheek	Yes (BM)	CWS, HR, subgroup H/CEVAIE	-
C100	8	m	Embryonal (botryoid)	Roof urinary bladder	No	CWS, SR, subgroup D	-
C102	14	f	Alveolar	Left upper arm	Yes (lung, skeletal, BM)	CEVAIE/TECC/rEECur	*PAX3/FOX01A*
C123	6	f	Alveolar with neurogenic component/ ectomesenchymoma	Right side abdomen	No	CWS VAIA III	*PAX7/FOX01A*

^a^Age at time of diagnosis.

^b^Classification according to the World Health Organization.

^c^Treatment protocol stratification according to the Cooperative Weichteilsarkom Studiengruppe (CWS) guidelines.

^d^Genetic alterations detected in routine clinical diagnostic workup.

*BM* bone marrow, HR high-risk, *SR* standard-risk.

### Patient-specific ultrasensitive ctDNA analysis

Based on whole exome sequencing (WES) of tumor and leukocyte DNA, we designed personalized sequencing panels detecting ten tumor-specific SNVs and used them for ctDNA analysis of 130 plasma samples in twelve patients with RMS (see Supplementary Data 1 for primer sequences, Supplementary Data 2 for all sequencing data, and Supplementary Data 3 for ctDNA data, including only the genomic positions of interest). A total of 634 SNVs (mean, 53; range, 16–145 SNVs/patient) with a variant allele frequency of >10% were detected by WES. Of these, nine SNVs were oncogenic mutations in genes recurrently altered in RMS (Supplementary Table 1; https://cancer.sanger.ac.uk/cosmic)^20,21^.

**Fig. 1:**
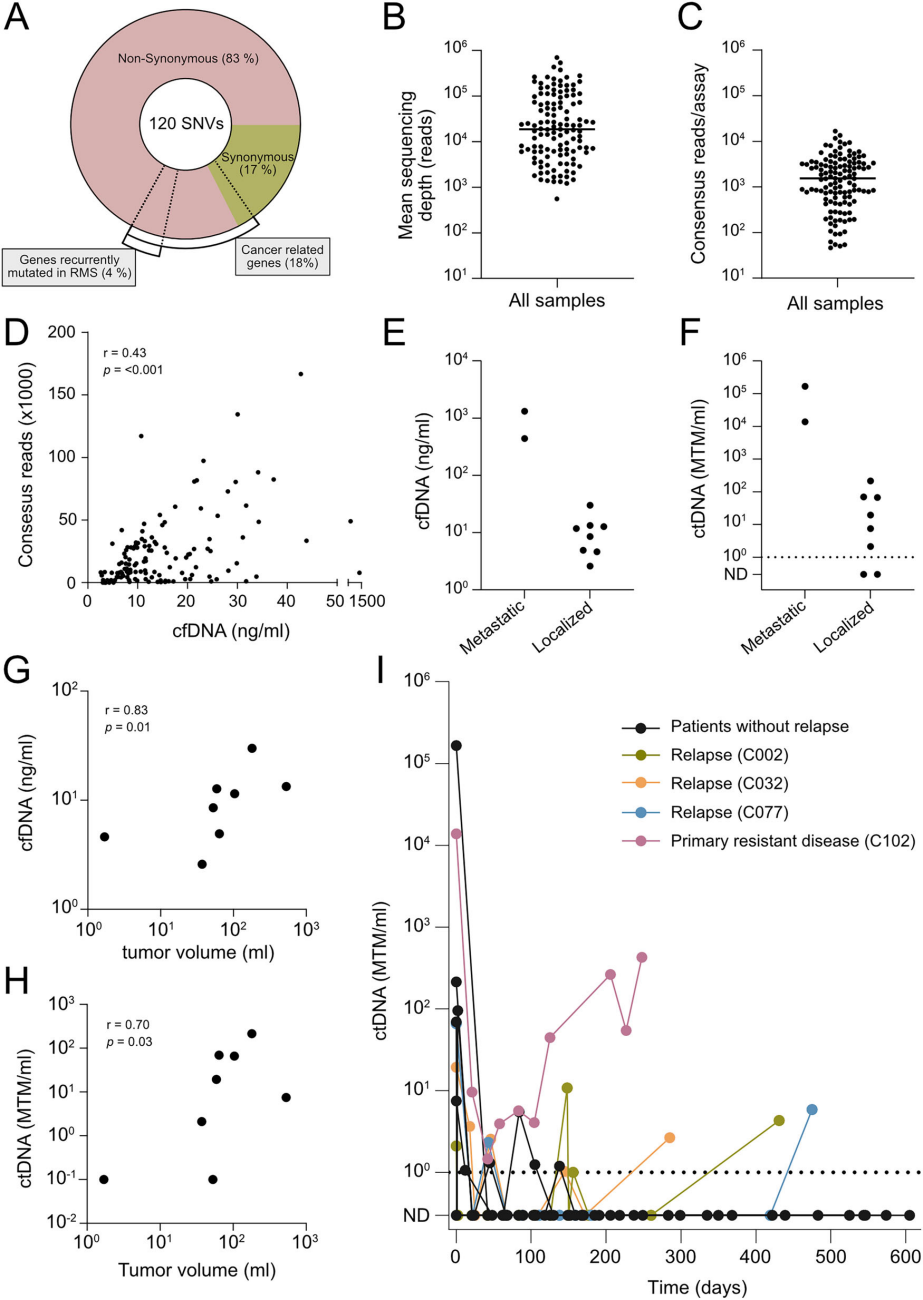
Tumor-informed ctDNA analysis for monitoring of tumor burden in RMS. **A** Proportion of targeted SNVs characterized as Synonymous and Non-Synonymous variants used in personalized ctDNA panels. **B** Sequencing depth. Each datapoint represents the mean number of raw reads at each SNV position (regardless of UMIs) for one plasma sample (*n* = 123). Median, 18,787. **C** Average number of consensus reads per assay and plasma sample (*n* = 123). Median, 1,537. **D** Correlation between the total number of consensus reads in ten assays/sample and cfDNA concentration/mL plasma, Spearman correlation coefficient is shown (*n* = 130). **E** Levels of cfDNA at time of diagnosis in children with metastatic (*n* = 2) and localized (*n* = 8) RMS. **F** Levels of ctDNA at time of diagnosis in children with metastatic (*n* = 2) and localized (*n* = 8) RMS. MTM was defined as a consensus read harboring a tumor-specific SNV. **G** Correlation between cfDNA concentration and tumor volume at diagnosis in patients with localized RMS (*n* = 8). The Spearman correlation coefficient is shown. **H** Correlation between ctDNA concentration and tumor volume at diagnosis in patients with localized RMS (*n* = 8). The Spearman correlation coefficient is shown. **I** Total levels of ctDNA over time in twelve RMS patients.

A majority (83%) of the 120 SNVs included in the panels were categorized as non-synonymous variants (Fig. 1A). Eighteen of the non-synonymous SNVs were found in genes relevant for cancer^22^, out of which five were oncogenic mutations in genes recurrently altered in RMS (*MYOD1* p.L122R, *NRAS* p.G12C, *FGFR4* p.V550L, *CTNNB1* p.K335I and *TP53* p.Y163C). The remaining four oncogenic mutations (including *MYOD1* p.L122R in patient C068) detected by WES were excluded from the sequencing panels due to technical difficulties with panel design.

The sequencing panels for the different patients were not overlapping, except for the inclusion of an identical SNV in *EMC7* for two patients (C090 and C102). The sequencing depth (median number of raw reads at each SNV position, regardless of UMIs) was 18,787 (Fig. 1B), and the median number of consensus reads was 1537 per assay (Fig. 1C). A positive correlation was seen between sample cfDNA concentration and total number of consensus reads (*r* = 0.43, *p* < 0.001) (Fig. 1D). Seven of the 130 plasma samples (5.4%) were excluded from downstream analysis due to low sequencing coverage (less than 400 consensus reads for all assays combined) leaving a median of ten successfully analyzed samples per patient (range 7–16).

Three (2.5%) of the selected SNVs were retrospectively identified as germline variants as they remained at consistent levels in plasma throughout the treatment (Supplementary Fig. 1), and were therefore excluded from the ctDNA analysis. Reviewing the WES of leukocyte DNA, two of the germline variants had low sequencing depth (15 and 16 reads, respectively), whereas the third variant showed one read harboring the mutation at a sequencing depth of 120 reads (Supplementary Fig. 2).

### cfDNA and ctDNA levels correlate with tumor burden at diagnosis

Plasma samples collected before treatment started were available from ten out of twelve patients. The two patients with metastatic disease at diagnosis (C090 and C102) showed considerably higher levels of cfDNA (median 876, range 439–1313 ng/mL) before treatment start compared to patients with localized disease (median 8.4, range 2.6–29.9 ng/mL) (Fig. 1E). In line with this, pre-treatment ctDNA levels were more than a thousand times higher in patients with metastasized disease (median 89,762, range 13,783–165,741 mutated tumor molecules (MTM)/mL) than in the localized tumor patient group (median 13.4, range 0.3–214.7 MTM/mL) (Fig. 1F). Among patients with localized RMS, the pre-treatment level of cfDNA and ctDNA correlated positively with tumor volume (*r* = 0.83, *p* = 0.01 and *r* = 0.70, *p* = 0.03 respectively), indicating that both parameters can be used as surrogate markers for tumor burden at the time of diagnosis (Fig. 1G, H). There was no clear difference in cfDNA and ctDNA levels at diagnosis between alveolar and embryonal RMS (Supplementary Fig. 3).

### ctDNA analysis for the detection of disease relapse

**Fig. 2:**
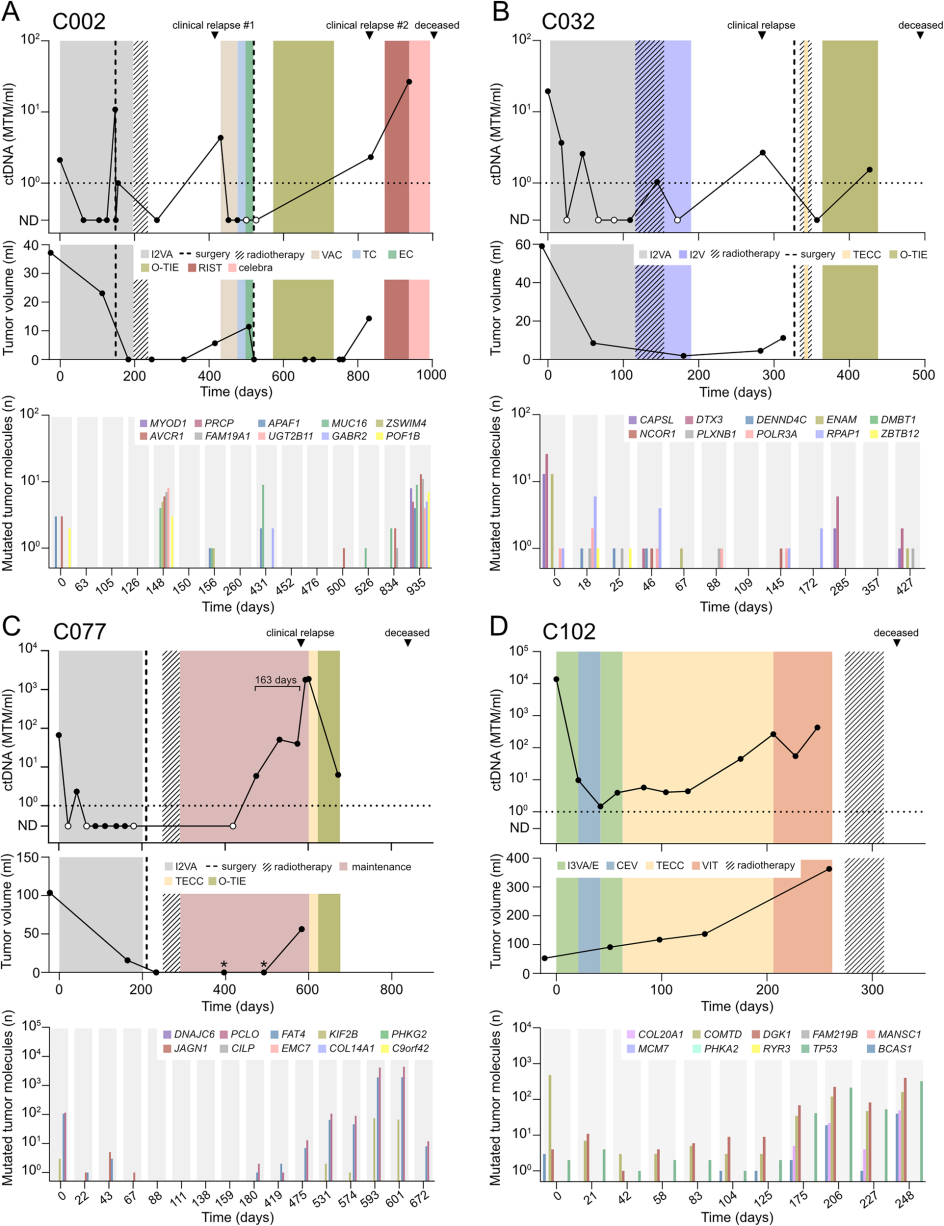
RMS relapse and disease progression are associated with elevated ctDNA levels. Levels of ctDNA (top), approximated tumor volume (middle), and number of mutated molecules for each individual targeted SNV over time (bottom) in patient **A** C002, **B** C032, **C** C077, and **D** C102. White circles indicate trace levels of ctDNA. Asterisks (C077) indicate dates of CT scans showing hypertrophy of the pleura and slightly enlarged lymph nodes in the area of the primary tumor, deemed as postoperative masses and/or nonspecific inflammation. I2VA Ifosfamide (two doses), Vincristine, and Actinomycin D, VAC Vincristine, Adriamycin, and Cyclophosphamide, TC Topotecan and Cyclophosphamide, EC Etoposide and Carboplatin, O-TIE Oral maintenance therapy with Trofosfamide, Idarubicine, and Etoposide, RIST Rapamycin, Irinotecan, Sprycel (dasatinib), and Temozolomide, I2V Ifosfamide (two doses) and Vincristine, TECC Topotecan, Etoposide, Cyclophosphamide, and Carboplatin, I3VA/E alternating courses of I3VA, Ifosfamide (three doses), Vincristine, and Actinomycin and I3VE, Ifosfamide (three doses), Vincristine, and Etoposide, CEV Carboplatin, Epirubicin, and Vincristine, VIT, Vinorelbine, Irinotecan, and Temozolomide.

Three of the patients experienced a total of four relapses, which were all associated with increased ctDNA levels (Fig. 1I). Patient C002 (Fig. 2A) presented at eight years of age with spindle cell RMS harboring *MYOD1* p.L122R. The tumor was located in the temporal region, causing local bone destruction but without signs of metastases. Surgery was performed after seven courses of chemotherapy, which was followed by two additional courses of chemotherapy as well as radiotherapy. Six months after the final treatment, the patient experienced a local relapse of the primary tumor that also progressed during the relapse therapy. After a second surgery and maintenance therapy, local progression was observed in the mandible and skull base with an intracranial component. The patient was transferred to third-line chemotherapy but passed away four months later due to disease progression. Both relapses were associated with an increase in ctDNA levels, with concentrations similar to or above the pre-treatment level. The level of ctDNA was relatively low at diagnosis, with only three out of ten SNVs identified, and became undetectable during neoadjuvant treatment. After seven courses of chemotherapy (day 148), ctDNA reappeared with the detection of six SNVs. At the time of the first relapse (day 431), three other SNVs were observed, none of which were present in the pre-treatment sample. During progressive disease after the second relapse (day 935) all ten SNVs were present at high levels in cfDNA. Of note, the oncogenic mutation *MYOD1* p.L122R was not found in cfDNA until the last analyzed timepoint. The detection of different SNVs at different phases of the disease underlines the benefit of using several mutations to enable sensitive ctDNA profiling.

Patient C032 (Fig. 2B) was diagnosed with alveolar RMS (*PAX3::FOXO1* positive) in the nasal cavity at ten years of age. Four months after the end of treatment, which included nine courses of chemotherapy and radiotherapy, a metastatic relapse was found in the brain and spine. After resection of a tumor at vertebra L2, the patient received palliative treatment including radiotherapy to the cerebellum and spine, one course of intravenous chemotherapy, and oral low-dose chemotherapy. The patient passed away seven months after the detection of the relapse due to progressive disease. In response to initial chemotherapy, the patient showed decreasing levels of ctDNA, which eventually became negative. However, ctDNA reappeared during radiotherapy and relapse was associated with increasing concentrations. Between one and five mutations were identified during initial chemotherapy (day 0 to day 88). Two tumor mutations were present in plasma at the time of clinical relapse (day 285), while four SNVs were detected at the last timepoint.

Patient C077 (Fig. 2C) was diagnosed with alveolar RMS (*PAX3::FOXO1* positive) in the thoracic wall at four years of age. Treatment was initiated with chemotherapy, followed by extended tumor resection and radiotherapy. Disease relapse in the primary tumor location was observed on a CT scan ten months into maintenance treatment, and the patient passed away 8 months after the start of relapse treatment. The patient responded well to initial treatment and became ctDNA-negative during neoadjuvant chemotherapy. However, ctDNA was detected in three consecutive samples starting 163 days before the clinical relapse. During this time period, the level of ctDNA increased by over a hundredfold, indicating significant tumor progression. Of note, CT scans performed 183 and 87 days before the relapse showed mild hypertrophy of the pleura and slightly enlarged lymph nodes in the area of the primary tumor. This was deemed as postoperative masses and/or nonspecific inflammation at the time, but was retrospectively considered to be a potential early sign of the upcoming relapse. Only three of ten SNVs were observed at any time point.

### ctDNA remains detectable throughout treatment in a patient with primary resistant disease

Patient C102 (Fig. 2D) was diagnosed with alveolar (*PAX3::FOXO1* positive) RMS in the *triceps brachii* at 14 years of age. A CT scan revealed lung metastases, and RMS cells were found in a bone marrow biopsy. An MRI after three courses of chemotherapy showed increased primary tumor size, particularly at a medial multilobulated component. 18-Fluoro-deoxyglucose positron emission tomography (FDG-PET) showed reduced uptake at the primary tumor site, but revealed signs of liver metastasis. The treatment was switched to second- and third-line chemotherapy, but the progression continued, and the patient passed away after receiving palliative radiotherapy towards the primary tumor site and surrounding lymph nodes. While the primary tumor was growing, the level of ctDNA was reduced during the first cycles of chemotherapy. This was in line with the reduced uptake on the PET scan and may reflect a mixed response, where some subclones of the disease are eradicated and others are resistant to treatment. However, unlike in all other patients, ctDNA never became negative but plateaued at the beginning of second-line treatment, followed by a stepwise increase during later disease progression. Four SNVs were detectable at diagnosis, and the same four SNVs, along with an additional one (a total of five SNVs), were present in the blood during late disease progression.

### ctDNA becomes undetectable upon successful treatment in RMS

**Fig. 3:**
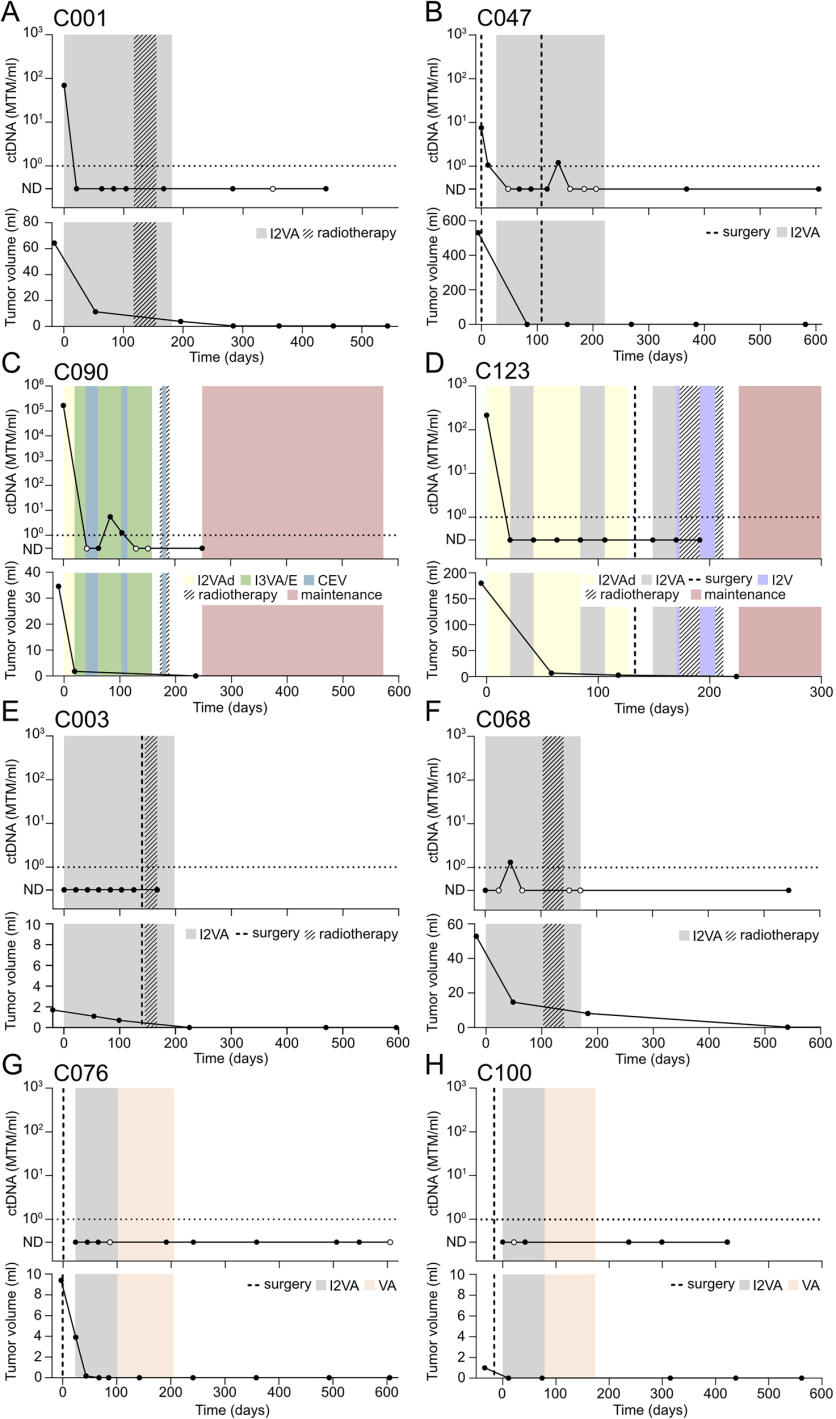
ctDNA levels show a stepwise decline upon successful treatment. Levels of ctDNA(top) and approximated tumor volume (bottom) over time in patient **A** C001, **B** C047, **C** C090, **D** C123, **E** C003, **F** C068, **G** C076, and **H** C100. I2VA Ifosfamide (two doses), Vincristine, and Actinomycin D, I3VA/E alternating courses of I3VA, Ifosfamide (three doses), Vincristine, and Actinomycin and I3VE, Ifosfamide (three doses), Vincristine, and Etoposide, CEV Carboplatin, Epirubicin, and Vincristine, I2VAd Ifosfamide (two doses), Vincristine, and Adriamycin, I2V, Ifosfamide (two doses) and Vincristine. White circles indicate trace levels of ctDNA.

Eight patients remained free from relapse during a median follow-up time of 42 months after the end of treatment (range 13–75 months). In four of them, ctDNA was detected at diagnosis and became negative during treatment (Fig. 3A–D). Two patients who had relatively small and localized tumors in the urinary bladder (C003) and below the left ear (C068) were ctDNA-negative at the time of diagnosis (Fig. 3E, F). In two patients (C076 and C100), the first plasma sample was collected after primary surgical removal of the tumor, which was macroscopically radical in both cases (Fig. 3G, H). All 16 plasma samples analyzed in these two patients were negative for ctDNA. In total, 14 plasma samples collected after the end of treatment in patients without disease relapse were all ctDNA-negative, suggesting a high specificity of the analysis as a tumor marker in these patients.

### Total cell-free DNA levels are not suitable for disease monitoring in RMS

In contrast to ctDNA levels, the concentration of total cfDNA did not consistently correlate with the clinical course of the disease in cases of disease recurrence or primary resistant disease (Supplementary Fig. 4A). Instead, cfDNA fluctuated between approximately 5 and 40 nanogram per milliliter of plasma during and after treatment also in patients who did not experience relapse of the disease (Supplementary Fig. 4B).

## Discussion

In this study, we designed individual sequencing panels targeting ten different SNVs customized for twelve children with RMS to monitor ctDNA levels at diagnosis, sequentially during treatment and in post-treatment follow-up samples. Using this tumor-informed approach, we show that ctDNA can be detected at diagnosis in most RMS patients, that ctDNA levels correlate with disease burden, and that disease recurrence is associated with increased ctDNA levels, which may be observed several months before clinical relapse.

ctDNA at the time of diagnosis was previously analyzed in RMS, and its presence has been suggested to predict clinical outcome^18,23,24^. Using a combination of ultralow passage whole-genome sequencing for copy number alterations (CNAs) and a custom sequencing assay for SNVs, ctDNA was observed in 57% of fusion-positive and 31% of fusion-negative RMS patients^18^, while another study using multiple detection methods confirmed ctDNA presence in 68% of pre-treatment RMS plasma samples^23^. By applying low-pass whole-genome sequencing of plasma cfDNA to detect CNAs, Christodoulou et al. found ctDNA in only 1 of 4 (25%) pre-treatment RMS samples^25^. Aiming at future ctDNA monitoring in all RMS patients, the previously reported moderate detection rates highlight the need for more sensitive methods. We evaluated multiple patient-specific markers simultaneously, which may improve the sensitivity without increasing the volume of blood used for the analysis. In support of this, only a subset of the mutations monitored appeared in many ctDNA-positive samples, and the combination of detected mutations varied over the course of treatment.

Previous studies of RMS have focused on oncogenic mutations as markers of ctDNA, leading to the exclusion of patients lacking the selected variants^14–17,19,24^. Traux de Wardin et al. applied a comprehensive methodology combining targeting of both translocation break points, SNVs and CNVs, but still had to select patients based on the occurrence of genetic alterations included in the sequencing panels^17^. Their custom RMS sequencing panel spanned a total region of 61 kb and included a median of two tumor-specific variants per sample. By utilizing individualized panels and including SNVs irrespective of biological significance, we analyzed ten markers of ctDNA per patient with a total sequenced region of only ~1 kb. All patients in our study were eligible for the analysis, and ctDNA was found at diagnosis in eight of ten children with available pre-treatment plasma samples.

Childhood tumors are associated with a low number of recurrent oncogenic mutations, and only nine SNVs considered to have biological relevance were detected by WES in all twelve patients combined. Therefore, the majority of SNVs included in our sequencing panels were passenger mutations, which could potentially increase the risk of losing individual markers over time due to tumor evolution. This was, however, not observed in our cohort, as all SNVs present at diagnosis in the four patients who experienced relapse or primary treatment resistance were also detected during late-stage disease. Our results support the use of passenger mutations as markers of ctDNA, although this requires confirmation in larger studies involving different malignancies.

The tumor-informed panels developed for this study yielded a median of 15,463 error-corrected consensus reads per sample. A minimum of four mutant reads (1 mutant molecule per mL of plasma based on cfDNA isolated from 4 mL of plasma) was required to report the sample as ctDNA positive, corresponding to a limit of detection (LOD) of approximately 0.026%. Our approach demonstrates a higher sensitivity compared to two previously reported methods for ctDNA detection in RMS, which achieved LODs of 3% using ultralow-passage whole-genome sequencing and 0.4% using SNV custom sequencing^18^. Although not yet validated for RMS, the commercially available tumor-informed test Signatera has demonstrated an LOD as low as 0.01% for ctDNA in several adult cancer types^26–28^. Our results suggest that such commercial tumor-informed sequencing panels might also be applicable in RMS, despite the lower frequency of oncogenic mutations as potential markers of ctDNA compared to adult malignancies.

Earlier recognition of relapse in RMS and, consequently, a more rapid initiation of relapse therapy may potentially improve the dismal prognosis for this patient group. Several previous studies have reported on increased plasma levels of ctDNA at the time of clinically apparent disease recurrence, but have been unable to detect a rise in ctDNA concentration prior to clinical observation^16,17,19^. In two separate RMS cases, tracking tumor-specific fusion genes in plasma (PAX3::FOXO1 in one patient and PAX3::NCOA1 in another) enabled detection of rising ctDNA levels 4 to 6 months prior to clinical relapse^14,15^. We observed increased ctDNA levels in all five cases of relapse or disease progression, and in one case, the ctDNA increase was observed 163 days before the clinical relapse. In this patient, CT scans performed 6 and 3 months before the diagnosis of relapse showed a mass at the site of tumor surgery, which was noted by the radiologists but was not considered as tumor growth. This is an example of when ctDNA analysis, if performed prospectively, could help to distinguish between a benign and malignant mass detected through radiology. All three patients who experienced relapse had relatively low (less than 100 MTM/mL) ctDNA levels at diagnosis, which supports a role for longitudinal ctDNA analysis also in such cases.

Although ctDNA was present at the time of clinical relapse in all cases, it was transiently undetectable during and after treatment in the patients who later had a relapse of their disease. This suggests that our method may not effectively detect minimal residual disease that persists at the end of therapy and ultimately leads to relapse, underscoring the need for longitudinal ctDNA analysis during follow-up. In the three cases of disease recurrence (two in patient C002 and one in patient C034) that became ctDNA-positive at the time of clinical relapse but not before, no plasma samples were available during six, ten and four months prior to the relapse, respectively. A relatively high frequency of sample collection during follow-up is recommended to achieve an early detection of disease relapse.

Temporary fluctuations in ctDNA were observed during treatment in three of the patients who remain disease-free (C047, C068, and C090). Although such fluctuations did not occur after the end of therapy, we cannot conclusively determine whether the observed increases represent true rises in tumor burden or are false-positive results. In a potential clinical setting, low levels of ctDNA should be interpreted cautiously until larger studies are available.

An important limitation of tumor-informed ctDNA analysis is that the method is unable to detect novel genetic alterations that may appear during or after treatment as potential drivers of disease progression. Therefore, if a relapse is detected using our approach, a broader standardized genetic analysis of tumor biopsy DNA or cfDNA may be useful to investigate the biology of the recurrent tumor.

Three of the 120 SNVs included in the sequencing panels were retrospectively ruled out as germline variants as they persisted at unchanged allele frequencies throughout the treatment, while the other SNVs in the panels reflected disease burden. These variants were associated with low sequencing depth or had one read harboring the mutation of interest in WES of germline reference DNA. In a potential clinical setting with prospective ctDNA analysis, including such false variants in tumor-informed sequencing panels could cause problematic errors since the germline nature of the variants may not be apparent in the first few analyzed samples. We recommend strict requirements regarding sequencing depth and the lack of reads harboring the mutation of interest in germline reference DNA to reduce the risk of including germline variants in tumor-informed sequencing panels.

Repeated imaging examinations are generally used for identification of relapse in RMS, although studies have failed to show a significant benefit of such surveillance programs^29–31^. Especially metastatic relapses affecting any part of the body can be challenging to find with radiology, which may be focused mainly on the primary tumor location. Further studies are needed to assess whether ctDNA analysis could replace repeated radiology examinations as a less invasive method for disease surveillance during follow-up in patients with RMS.

In the patient with primary resistant disease (C102), ctDNA levels showed a stepwise reduction during initial treatment despite increasing tumor volume. The declining ctDNA levels were in line with a positron emission tomography (PET) scan, which showed reduced intensity at the primary tumor site after three courses of chemotherapy. This suggests that tumor-informed ctDNA analysis may be a complement to imaging, as it provides information about the total burden of malignant cells rather than tumor size alone. However, as ctDNA levels initially decreased, monitoring ctDNA showed limited value for early detection of primary treatment resistance in this case. Patient-specific analysis also requires a certain time for design and validation of sequencing panels, which may further limit the use of the method during the first weeks of treatment.

With a few exceptions, such as neuron-specific enolase, chromogranin A, and urine catecholamine metabolites in neuroblastoma, alpha-fetoprotein (AFP) in hepatoblastoma, and human chorionic gonadotropin and AFP in germinomas, the use of biomarkers representing tumor burden in childhood malignancies is limited^32–35^. Currently, no liquid biomarkers for disease monitoring of RMS and other childhood sarcomas are clinically implemented. Our approach enabled sensitive ctDNA analysis in all twelve patients, even though well-defined genetic markers such as *PAX::FOXO1* rearrangements and *MYOD1* mutations were only identified in six of them. We demonstrate that tumor-informed ctDNA analysis may be used to monitor tumor burden over time in RMS and that patient-specific multitarget sequencing panels can be sensitive enough to detect disease relapse months before it is diagnosed using standard radiology examinations. Although the results are promising, validation in larger, prospective studies is needed before clinical application can be considered. Moreover, tumor-informed ctDNA analysis presents several challenges, including the requirement for readily accessible data on tumor genetics, streamlined panel design, and fast sample processing. These issues must be addressed to effectively evaluate the clinical utility of our approach for monitoring patients with RMS.

## Methods

### Patient recruitment and blood sampling

Twelve children with RMS diagnosed between March 2017 and September 2019 at Sahlgrenska University Hospital (SU) were included in the study (see Supplementary information for clinical case summaries). Written informed consent was obtained from the parents or legal guardians of all patients. The study was performed in accordance with the Declaration of Helsinki, and was approved by the regional ethical review board in Gothenburg (Ref. No. 655-17) and by the Swedish ethical review authority (Ref. No. 2019-06285). The patients were treated according to the Cooperative Weichteilsarkom Studiengruppe (CWS) guidance protocol version 1.6. Blood (~ 8.5 mL) was collected in cf-DNA/cf-RNA Preservative Tubes (Norgen Biotech). Samples at the time of diagnosis and during treatment were drawn via a central venous catheter or port-a-cath, whereas follow-up samples collected after removal of the central line were taken by peripheral vein puncture. All blood sampling was coordinated to align with routine clinical sampling schedules.

### Extraction of cell-free DNA from plasma

Blood samples stored at room temperature were centrifuged for 20 min at 420 x *g* within seven days from collection. The plasma was collected in XLX2000-2D Biobanking tubes (LVL Technologies) using a Freedom EVO liquid handling robot (Tecan), before storage at –80 °C. Cell-free DNA (cfDNA) was extracted from ~4 mL of thawed plasma using the QIAamp Circulating Nucleic Acid Kit (Qiagen) and was quantified using the Qubit dsDNA HS Assay Kit (ThermoFisher Scientific). Plasma samples from patients C001 and C002 were centrifuged for 20 min at 4500 x *g* prior to DNA extraction. All samples with low DNA concentration (< 7.5 ng/µL) were concentrated to a volume of 10–14 µL using Vivacon 500 with a molecular weight cut-off of 30 kDa (Sartorius).

### Patient-specific ctDNA-assay design

Tumor DNA was extracted from formalin-fixed paraffin-embedded (FFPE) tissue remaining after the diagnostic procedure using the GeneRead FFPE DNA kit (QIagen). Cellular non-cancer reference DNA was extracted from the cellular component of the blood sample using the DNeasy blood and tissue kit (Qiagen). Libraries were prepared using the SureSelectQXT target enrichment kit (Agilent Technologies), and WES was performed using the NextSeq 500 system with a NextSeq 500 v2 reagent kit (Illumina) at the Clinical Genomics Gothenburg, SciLifeLab, Gothenburg, Sweden.

Mapping to the reference genome, duplicate removal and variant calling were performed using the open-source bioinformatic pipeline Sarek^36^. The variant callers FreeBayes, Mutec2, and Strelka were used for SNV detection and removal of germline variants. Ten tumor-specific SNVs per patient were selected as targets for a personalized multiplex PCR panel. The SNVs were selected based on tumor variant allele frequency and in a few cases possible pathological relevance (one SNV (*NCOR1*) in patient C032 and three SNVs (*CTNNA2*, *SF3B3* and *TEC*) in patient C047), requiring an alternate variant read depth of ≥10, detection of maximum one variant read in germline DNA, and passing manually review using Integrative Genomic Viewer (https://igv.org). Primers were designed using Primer-BLAST^37^, at a 60 °C or 62 °C annealing temperature with amplicons sized from 75 to 105 bp.

### Library preparation and cfDNA sequencing

To enable correction of polymerase-induced errors and uneven amplification, library preparation was performed using “Simple multiplexed PCR-based barcoding of DNA for ultrasensitive mutation detection by next generation sequencing” (SiMSen-Seq)^38^. SiMSen-Seq library preparation is performed in two PCR steps (barcoding and adapter PCR) and includes the addition of universal SiMSen-Seq oligonucleotides, including a unique molecular identifier (UMI) to all target forward primers. Barcoding and adapter PCR were performed in a total reaction volume of 15 µL and 60 µL, respectively. The DNA polymerase was inactivated by the addition of 45 ng/ul of protease (Streptomyces griseus, Sigma Aldrich) dissolved in 30 µL TE buffer, pH 8.0 (Ambion, Thermo Fisher Scientific), at the beginning of the 65 °C step of the barcoding PCR (Table S2).

The SiMSen-Seq libraries were evaluated using an HS NGS Fragment Kit on a 5200 Fragment Analyzer and analyzed in Prosize data analysis software (all Agilent Technologies). Libraries were purified using Pippin Prep Cassette 2%, 100–600 bp, Internal Marker (Sage Science) with the target range of 205–300 bp or the Agencourt AMPure XP system (Beckman Coulter). Library pools were sequenced on either the MiniSeq or NextSeq 1000 platforms using MiniSeq Mid Output Kit (300 cycles), MiniSeq High Output Kit (150 cycles), or NextSeq 500/550 Mid Output Kit v2.5 (150 Cycles) (all Illumina) with loading concentrations between 1.2 and 1.8 pM, 10–20% PhiX (Illumina), and single-end reads with 150 bp mode.

### Bioinformatical analysis

Raw sequencing reads were bioinformatically processed using UMIErrorCorrect^39^, including alignment to the Human build 38 reference genome and clustering into UMI families according to target DNA regions. Sequencing reads were collapsed into error-corrected consensus reads, requiring a family size of at least three reads per UMI. A mutated tumor molecule (MTM) was defined as a consensus read harboring a tumor-specific SNV. The ctDNA level was determined by the total number of MTM per milliliter of plasma in all assays combined, with samples containing at least 1 MTM/mL classified as ctDNA-positive. Since SiMSen-Seq generates an average of two barcodes per DNA molecule, the plasma concentration of the original mutated DNA molecules is roughly half of the MTM value.

### Tumor volume evaluation

Tumor diameter measurements in three dimensions (d1–d3) were collected from radiology reports of MRI, CT and in one case (C076), ultrasonography examinations. Tumor volume was approximated using the volume of an ellipsoid formula (*V* = 1/6 x *π* x d1 x d2 x d3). When only two diameter measurements were available, the third diameter was assumed to be the average of the two known measurements. For tumors with only one available diameter measurement, the other two diameters were calculated based on the presumption of a spherical tumor shape. In one case (C047), the pathologist’s length measurements after radical resection were used to estimate tumor volume, due to difficulties in delineating intracranial tumor margins.

### Statistical analysis

GraphPad Prism version 10.4 (Dotmatics) was used for statistical analysis and graphical visualization. Statistical tests used are specified in the figure legends.

## Supplementary information

Supplementary Information

Supplementary Data1

Supplementary Data2

Supplementary Data3

## Data Availability

All relevant data used to perform the analysis are available within the article or the Supplementary Information. Primer sequences are found in Supplementary Data 1, bioinformatically analyzed sequencing data are found in Supplementary Data 2, and fully processed ctDNA data are found in Supplementary Data 3. Clinical information for each case is provided in Supplementary Information, pages 7–11, and is summarized in Table 1. Whole exome sequencing data of tumor and germline DNA, and raw data from cell-free DNA sequencing, are not available to the public to protect the privacy and confidentiality of the patients, but can be requested from the corresponding author for academic use only. Such requests will be reviewed within four weeks to determine whether the request is subject to confidentiality obligations. Any data shared will be de-identified, and secondary use will be strictly prohibited.

## Code availability

All cell-free DNA sequencing data were analyzed using the open-source bioinformatic pipeline UMIErrorCorrect (https://github.com/tobbeost/umierrorcorrect).
