# Supplementary material for: Patient-specific sequencing panels enable sensitive circulating tumor DNA analysis in rhabdomyosarcoma independent of genetic profile
Source: NPJ Precis Oncol. 2025 Oct 21;9:327. doi: 10.1038/s41698-025-01147-6 (PMC12540861; doi:10.1038/s41698-025-01147-6)
Supplement: Supplementary file 1 — Supplementary Information [file 41698_2025_1147_MOESM1_ESM.pdf]

Supplementary information

Patient-specific sequencing panels enable sensitive circulating tumor DNA analysis in rhabdomyosarcoma independent of genetic alterations

Rahmqvist and Dahlstrand Rudin et al.

Supplementary Data 1. Primer sequences – Excel file.

Includes the sequences of all primers used in the patient-specific sequencing panels. Fw, forward; rev, reverse.

Supplementary Data 2. All sequencing data – Excel file.

Includes cell-free DNA sequencing data processed with UMIErrorCorrect, shown as number of consensus reads. Each consensus read consists of at least three unique reads with the same UMI. See the "Descriptions" sheet for information about each column.

Supplementary Data 3. ctDNA data – Excel file.

Includes ctDNA data focused only on the sites of the SNVs included in the patient-specific panels, as well as cfDNA concentration and input for each timepoint. See the "Descriptions" sheet for information about each column.

Supplementary Table 1. Mutations detected by WES.

Patient	Number of SNVs with VAF >10%	Oncogenic mutations	Tumor DNA VAF (%)	Included in panel (yes/no)
C001	97	*FGFR4* p.V550L	66	yes
		CTNNB1 p.K335I	19	yes
C002	35	*MYOD1* p.L122R	98	yes
C003	37			
C032	24			
C047	48	*NRAS* p.Q61H	64	no*
C068	35	*MYOD1* p.L122R	75	no*
C076	84	*NRAS* p.G12C	80	yes
C077	43			
C090	45			
C100	16	*TP53* p.H179D	44	no*
		*DICER*1 p.E1813G	37	no*
C102	145	*TP53* p.Y163C	87	yes
C123	25			

*Excluded due to technical problems with panel design.

Supplementary Table 2. SiMSen-Seq protocol.

Barcoding PCR

Reagent	Manufacturer	Concentration	PCR protocol		
Platinum SuperFi buffer	Thermo Fisher Scientific	x1	*Temperature*	*Time*	*Cycles*
dNTP mix	Sigma-Aldrich	0.2 mM	98 ˚C	3 min	1
L-Carnitine	Sigma-Aldrich	0.5 M	98 ˚C	10 sec	3
Barcoding primer		40 nM	60/62 ˚C	6 min	
Platinum SuperFi polymerase	Thermo Fisher Scientific	0.01 U/µl	72 ˚C	30 sec	
Nuclease-free water		–	65 ˚C	15 min	1
cfDNA		up to 40 ng	95 ˚C	15 min	1

Adapter PCR

Reagent	Manufacturer	Concentration	PCR protocol		
Q5 High-Fidelity Mastermix	New England Biolabs	x1	*Temperature*	*Time*	*Cycles*
Reverse adapter primer	Integrated DNA Technologies	400 nM	98 ˚C	3 min	1
Universal forward primer	Integrated DNA Technologies	400 nM	98 ˚C	10 sec	27 (0.2 ˚C/s)
Nuclease-free water		–	80 ˚C	1 sec	
Product from barcoding			72 ˚C	30 sec	
			76 ˚C	30 sec	

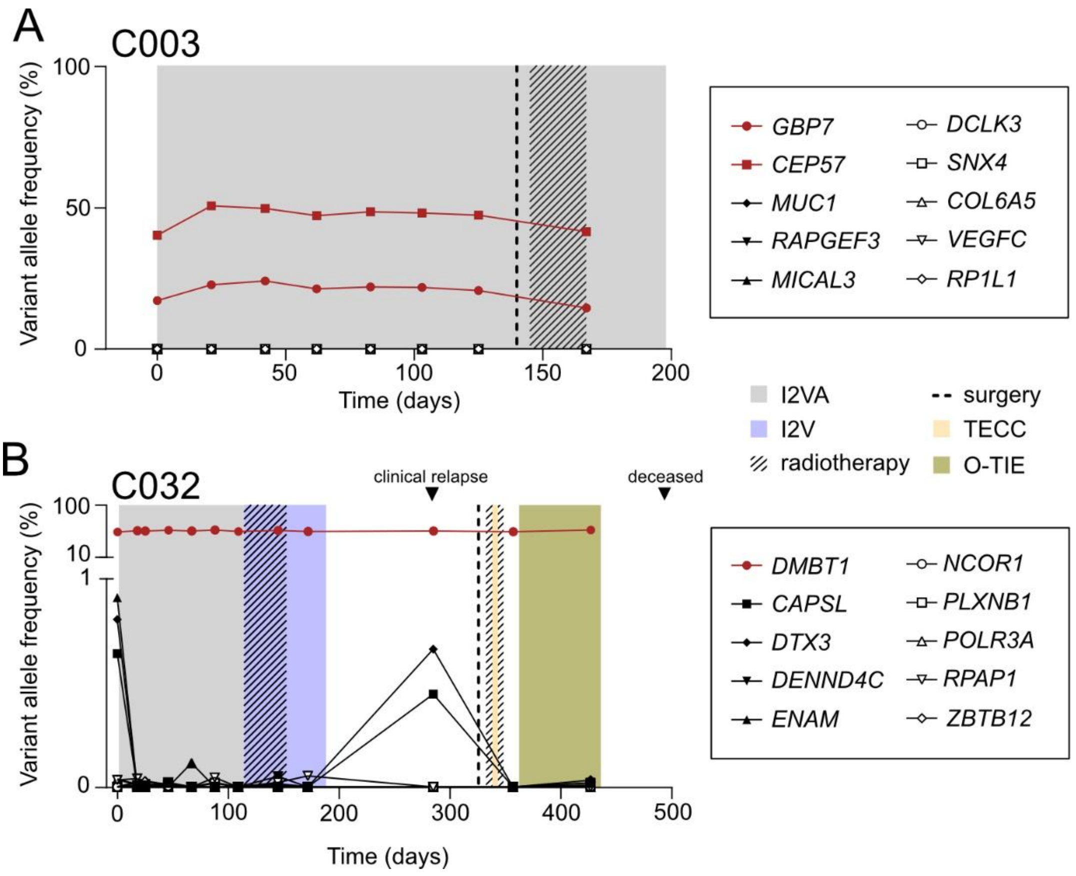

Supplementary Fig. 1. Mutations presenting with stable allele frequencies over time were assessed as germline. Allele frequencies (%) over time for ten individual SNVs in patient C003 **(A)** and C032 respectively **(B)**. Red symbols indicate variants assessed as germline. I2VA, Ifosfamide (two doses), Vincristine, and Actinomycin D; I2V, Ifosfamide (two doses) and Vincristine; TECC, Topotecan, Etoposide, Cyclophosphamide, and Carboplatin; O-TIE, Oral maintenance therapy with Trofosfamide, Idarubicine, and Etoposide.

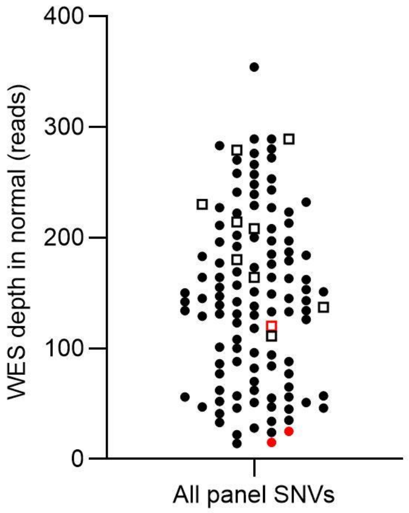

Supplementary Fig. 2. Variants retrospectively assessed as germline presented with few or alternate reads in normal sample WES. WES read depth in normal sample for all SNVs included in sequencing panels. Red symbols indicate mutations assessed as germline. Squares represent SNVs presenting with one alternate read in normal, while no alternate reads were seen for the remaining SNVs.

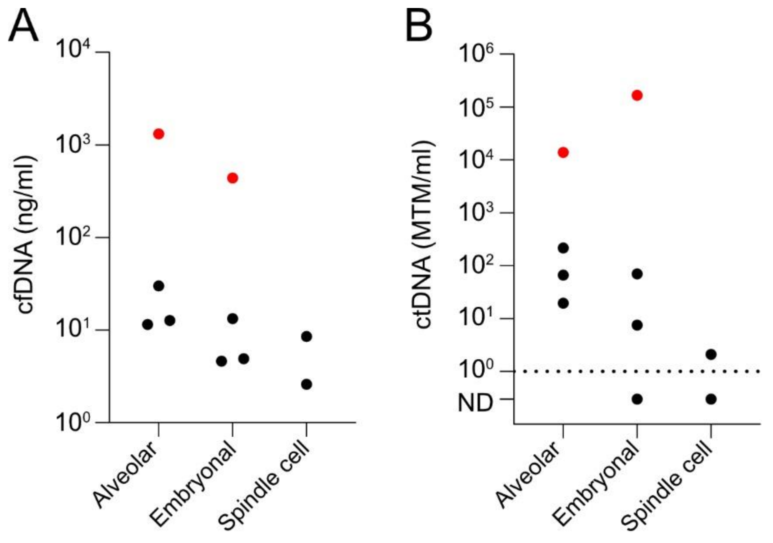

Supplementary Fig. 3. Levels of cfDNA and ctDNA at diagnosis in RMS subtypes. Levels of cfDNA **(A)** and ctDNA **(B)** at time of diagnosis in children with RMS, separated by disease subtypes. Red symbols indicate patients with metastatic disease at diagnosis.

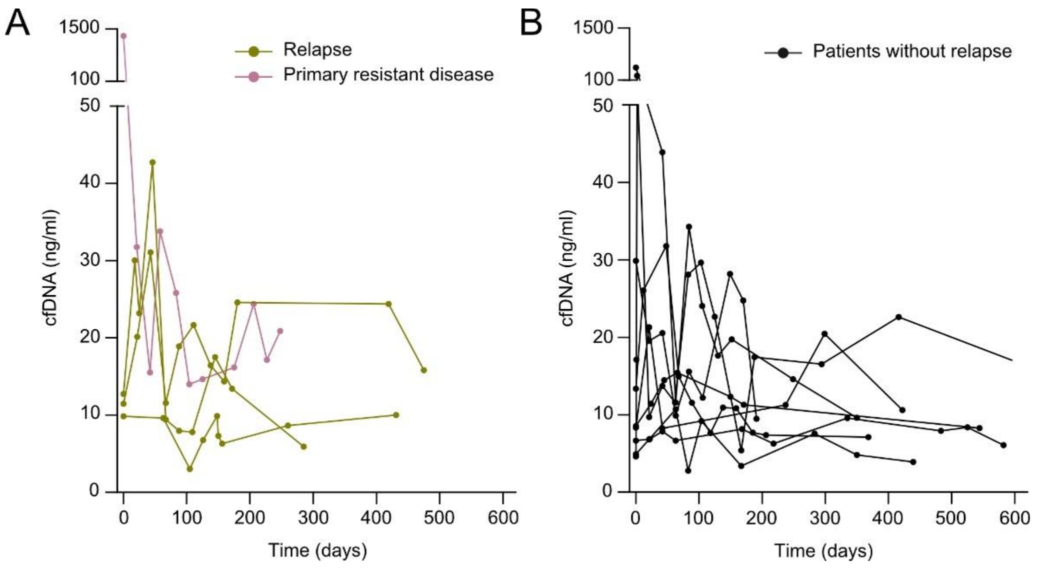

Supplementary Fig. 4. cfDNA levels do not consistently correlate with the course of disease in RMS patients. (A) Plasma levels of cfDNA over time in three patients experiencing disease relapse (green) and one patient presenting with primary resistant disease (pink). **(B)** Plasma levels of cfDNA over time in eight successfully treated patients.

Clinical case summaries

Patient C001. The patient presented at nine years of age with embryonal RMS in the left cheek, measuring 4.2 x 5.9 x 5.3 centimeters. There was no evidence of metastasis. Treatment was given according to the Cooperative Weichteilsarkom Studiengruppe (CWS) Guidance protocol, standard risk, subgroup C, which included nine cycles of Ifosfamide (two doses), Vincristine, and Actinomycin D (I2VA). The tumor was deemed to be unresectable and local treatment was given with proton beam radiotherapy in parallel with chemotherapy cycles seven and eight. The patient remained free from relapse at the last clinical follow-up two years and three months after the end of treatment.

Patient C002. The patient was diagnosed at eight years of age with spindle cell RMS of botryoid type in the left temporal region, measuring 4 x 3.5 x 5.5 centimeters. The tumor caused local bone destruction but there were no metastases. Treatment was given according to CWS guidance, subgroup E. After seven out of nine I2VA courses, the patient underwent surgery which was macroscopically radical but microscopically viable tumor cells were seen in the resection margins. Following chemotherapy, the patient received proton beam radiotherapy (50.4 Gray). The patient was then randomised to the observational arm of the CWS 2007 high-risk protocol. Six months after completion of therapy a local relapse was seen on magnetic resonance imaging (MRI) at the site of the primary tumor, measuring 1.5 x 2.4 x 3 centimeters. No signs of metastases were found. The patient was started on relapse therapy with topotecan and carboplatin. An MRI two months into treatment showed progress of the tumor and treatment was changed to etoposide and carboplatin. The patient was re-operated with partial maxillectomy and resection of tumor in the infratemporal fossa. Suspected tumor cells were seen in the resection margin. After five months of oral maintenance therapy with trophosphamide, idarubicin and etoposide, the patient developed tenderness and swelling in front of the left ear and an MRI showed new tumors around the mandible, towards the skull base, and with an intracranial component growing through the oval window.

A new biopsy was performed and molecular analysis revealed a *MYOD1* mutation and overexpression of *ABL2*. The patient was then started on alternating cycles of sirolimus/dasatinib and irinotecan/temozolamide according to the RIST protocol, but the tumor continued to grow visibly during this treatment and the patient passed away four months later.

Patient C003. The patient presented at one year of age with hematuria, and an MRI showed a lobulated tumor in the floor of the urinary bladder measuring 1.7 x 1.9 x 2.5 centimeters. A biopsy through cystoscopy showed embryonal rhabdomyosarcoma. No evidence of metastases was found and treatment according to CWS guidance subgroup D was started. MRI after three courses of I2VA showed a 20 % reduction in tumor size. After seven (out of nine) courses of I2VA, the patient underwent surgery with a resection of the tumor and insertion of brachytherapy rods which remained for ten days. The patient

remained free from relapse at the latest clinical follow-up six years and five months after the end of treatment.

Patient C032. The patient presented at ten years of age with headache, epistaxis and blurred vision in the left eye. Radiology examinations showed a tumor measuring 4 x 5.5 x 5.5 centimeters located in the nasal cavity and the ethmoidal cells, invading the anterior cranial fossa and the left orbita. Biopsy showed alveolar rhabdomyosarcoma. No metastases were found and treatment according to CWS guidance, high risk, subgroup G was started (six courses of I2VA followed by three courses of ifosfamide and vincristine (I2V). Radiotherapy was given in parallel with chemotherapy cycle seven and eight, with a combination of proton and foton therapy to a total of 50.4 Gray. An MRI after completed treatment showed remaining contrast-enhancing tissue of uncertain origin. A positron emission tomography (PET) scan was done suggesting it not to be malignant. Four months after the end of treatment, MRI revealed three new intracranial lesions. The patient also had pain in the right hip, and an MRI one month later showed multiple metastases in the spine, of which the largest was located at verterbra L2. The patient then underwent resection of the metastasis at the L2 level. Analysis of the resected tissue showed it to consist of the original alveolar rhabdomyosarcoma.

Palliative radiotherapy was given to the cerebellum with 30 Gray and to the spine with 20 Gray, in combination with one course of topotecan, etoposide, cyclophosphamide and carboplatin (TECC). After this, oral maintenance therapy with alternating ten-day courses of trofosfamide/idarubicin and trofosfamide/etoposide was given for two and a half months. The patient then suffered a cerebral bleeding originating from one of the metastases and passed away less than two months after the end of treatment.

Patient C047. The patient presented at 15 years of age with a tumor in the left testis measuring 14 x 6.5 centimeters. A left-sided orchidectomy was performed and pathology analysis showed an embryonal rhabdomyosarcoma. The excision was macroscopically radical but microscopically a few tumor cells were seen in the pseudo capsule of the tumor. There was no evidence of metastases. Treatment according to CWS guidance, subgroup C, was started. After four (out of nine) courses of I2VA a hemiscrotectomy was performed as the initial surgery had not been microscopically radical. The patient remained free from disease at the latest follow-up visit six years after the end of treatment.

Patient C068. The patient presented at 12 years of age with a swelling below the left ear. ACT scan showed a soft-tissue tumor measuring 3.0 x 6.0 x 5.6 centimeter that dislocated the mandible forwards, and a biopsy showed a spindle-cell/sclerosing rhabdomyosarcoma of non-alveolar type harboring the *MYOD1* p.L122R mutation. No evidence of metastasis was found. Treatment was started according to CWS Guidance, subgroup E. The patient received nine courses of I2VA chemotherapy and proton beam radiotherapy to 50.4 Gray which was given in parallel with course six and seven.

MRI after the end of treatment showed a residual tumor with slight contrast-enhancement. The patient was monitored with MRI every third month during the first year after completing therapy, showing a stepwise size reduction of the residual tumor. At the latest follow up, four years and ten months after the end of treatment, the patient was in good clinical condition and showed no signs of disease relapse.

Patient C076. The patient presented at 15 years of age with a tumor in the right side of the scrotum measuring 2.5 x 3 centimeter. A right-sided orchidectomy was performed and pathology analysis showed a paratesticular embryonal rhabdomyosarcoma. No metastases were found. The tumor appeared to have been radically excised but with a narrow margin. Treatment according to CWS guidance, subgroup B, was started and the patient was given four courses of I2VA followed by five courses of vincristine and actinomycin D only. Follow-up ultrasound examinations have not shown any sign of recurrence and the patient remained well at the latest follow-up six years after the end of treatment.

Patient C077. The patient presented at four years of age with a lump on the left side of the anterior chest wall. A CT scan revealed a tumor in the thoracic wall measuring 7.0 x 4.2 x 6.1 centimeters, with an adjacent tumor measuring 1.5 x 2.0 x 2.0 centimeters. A needle biopsy showed alveolar rhabdomyosarcoma. There was no evidence of metastasis. The patient was treated according to CWS guidance high risk, subgroup G. Evaluation after three courses of I2VAshowed good response. After completion of nine courses of I2VA, the patient underwent extended tumor surgery, replacing the resected tissue with a Goretex plate supported by a horizontal metal wire. Viable tumor cells were seen in the resection margins. The patient then received radiotherapy up to 50.4 Gray toward the primary tumor site, followed by maintenance therapy with daily oral cyclofosfamide and weekly vinorelbin. After three and six months of maintenance therapy, CT scans showed a new elliptic structure in the location of the original tumor which was considered to be fluid in connection with the Goretex plate.

A new CT after nine months of maintenance therapy indicated a relapse at the site of primary tumors, which was confirmed with a needle biopsy. The patient received relapse treatment with topotecan, etoposide, cyclophosphamide and carboplatin (TECC), which was switched to oral treatment with trofosfamide, etoposide and idarubicin (O-TIE) after only one course of intravenous chemotherapy because of a severe neutropenic infection. The patient passed away seven months later due to progressive disease.

Patient C090. The patient presented at four years of age with a lump in the right cheek. A CT scan showed a tumor measuring 3.0 x 4.4 centimeters which surrounded the mandible and caused bone erosion, as well as a locoregional pathological lymph node and potential metastases in multiple cervical vertebrae. A tissue biopsy showed embryonal rhabdomyosarcoma. Treatment was started according to CWS guidance very high risk, subgroup H with ifosfamide, vincristine and doxorubicin. However, the bone marrow biopsy showed malignant cells leading to a change of treatment to the CEVAIE protocol, which includes cisplatin, etoposide, vincristine, actinomycin D, ifosfamide, and epirubicin. Evaluation

after four chemotherapy courses showed good response with significantly reduced contrast-enhancement, no vertebral tumors, and no malignant cells in the bone marrow biopsy. The patient then received five additional courses of chemotherapy and proton beam radiotherapy up to 50.4 Gray towards the primary tumor site. After that, MRI showed no sign of disease and the patient received maintenance therapy with daily oral cyclofosfamide and weekly vinorelbin for ten months. The patient remained free from relapse at the latest clinical follow-up three years and seven months after the end of treatment.

Patient C100. The patient presented at eight years of age with hematuria and CT showed a tumor in the roof of the urinary bladder measuring one centimeter. The tumor was removed by surgery and pathology analysis showed embryonal rhabdomyosarcoma of botryoid type. The surgery was macroscopically radical, but this could not be verified microscopically as the tumor fell apart during the operation. There was no evidence of metastasis. The patient was treated according to CWS guidance, subgroup D, starting with four courses of I2VA. Evaluation with cystoscopy (including tissue biopsies). and MRI after three courses showed no evidence of disease, and the treatment was changed to subgroup A. The patient then received five courses of vincristine and actinomycin D only. The patient remained free from relapse at the latest follow-up four years after the end of treatment.

Patient C102. The patient presented at 14 years of age with a lump in the left upper arm. MRI showed a heterogenous tumor measuring 5.0 x 2.7 x 7.0 centimeters in *triceps brachii*. Multiple metastases were found in the axillary lymph nodes, skeleton (pelvis, sacrum, and several vertebrae), lungs, and bone marrow. Tumor biopsy showed alveolar rhabdomyosarcoma. Treatment was started according to the CEVAIE protocol for primary metastatic disease. Response evaluation after three courses of chemotherapy showed that the tumor had increased in size, particularly a medial multilubolated component. PET scan showed reduced uptake in the primary tumor but also revealed liver involvement that was not seen before the start of treatment. Due to progressive disease, treatment was changed to the TECC protocol and the patient received six courses of topotecan, etoposide, cyclofosfamide and carboplatin. Evaluation after TECC showed a mixed response with progress of the primary tumor, axillary lymph nodes, and lung metastases, but PET uptake in the liver was no longer seen. The treatment was then switched to the rEECur protocol, starting with vincristine, irinotecan and temozolamide (VIT). After three VIT courses an MRI showed further increase in size and multiple necrotic areas in the primary tumor, as well as disease progress in the lungs and axillary lymph nodes. The patient then received palliative radiotherapy in the primary tumor area and left axilla to relieve pain. Less than two months later, the patient passed away due to progressive disease.

Patient C123. The patient presented at six years of age with a rapidly growing lump on the right side of the abdomen. MRI showed a retroperitoneal paravertebral tumor growing out towards the thoracic/abdominal wall. There was tumor growth into several intervertebral foramina but not into the spinal canal. The tumor surrounded the aorta and the vena cava inferior but did not compress the vessels.

Needle biopsies showed alveolar rhabdomyosarcoma. The tumor had a component with neurogenic differentiation and was classified as an ectomesenchymoma. No evidence of metastases was found and treatment was started according to CWS guidance VAIA III protocol. Evaluation after three courses of chemotherapy showed a very good response with only a thin layer of tumor tissue remaining. After six courses of chemotherapy the patient underwent tumor surgery with resection of part of the thoracic wall. Analysis of resected tissue showed between 10 and 50 % viable tumor cells, classifying the patient as a poor responder. In total, the patient received a total of nine chemotherapy courses, and radiotherapy towards the primary tumor site given concomitantly with the last two of them. The patient then started maintenance treatment with oral cyclophosfamide daily and intravenous vinorelbine weekly for 12 months. The patient remained free from relapse at the latest clinical follow-up three years and two months after the end of treatment.
